# A simple, sensitive, and low‐cost FACS assay for detecting antibodies against the native SARS‐CoV‐2 spike protein

**DOI:** 10.1002/iid3.446

**Published:** 2021-05-12

**Authors:** Julia Hambach, Tobias Stähler, Thomas Eden, Dorte Wendt, Natalie Tode, Friedrich Haag, Eva Tolosa, Marcus Altfeld, Anahita Fathi, Christine Dahlke, Marylyn M. Addo, Stephan Menzel, Friedrich Koch‐Nolte

**Affiliations:** ^1^ Institute of Immunology University Medical Center Hamburg‐Eppendorf Hamburg Germany; ^2^ Department of Virus Immunology Heinrich Pette Institute, Leibniz Institute for Experimental Virology Hamburg Germany; ^3^ Section Infectious Diseases, I. Medical Clinic and Polyclinic University Medical Center Hamburg‐Eppendorf Hamburg Germany; ^4^ Department of Clinical Immunology of Infectious Diseases Bernhard‐Nocht‐Institute for Tropical Medicine Hamburg Germany; ^5^ German Center for Infection Research, Partner Site Hamburg‐Lübeck‐Borstel‐Riems Hamburg Germany

**Keywords:** antibodies, flow cytometry, infectious diseases, virology

## Abstract

**Background:** Hamburg is a city state of approximately 1.9 Mio inhabitants in Northern Germany. Currently, the COVID‐19 epidemic that had largely subsided during last summer is resurging in Hamburg and in other parts of the world, underlining the need for additional tools to monitor SARS‐CoV‐2 antibody responses.

**Aim:** We aimed to develop and validate a simple, low‐cost assay for detecting antibodies against the native coronavirus 2 spike protein (CoV‐2 S) that does not require recombinant protein or virus.

**Method:** We transiently co‐transfected HEK cells or CHO cells with expression vectors encoding CoV‐2 S and nuclear GFP. Spike protein‐specific antibodies in human serum samples bound to transfected cells were detected with fluorochrome conjugated secondary antibodies by flow cytometry orimmunofluorescence microscopy. We applied this assay to monitor antibody development in COVID‐19 patients, household contacts, and hospital personnel during the ongoing epidemic in the city state of Hamburg.

**Results:** All recovered COVID‐19 patients showed high levels of CoV‐2 S‐specific antibodies. With one exception, all household members that did not develop symptoms also did not develop detectable antibodies. Similarly, lab personnel that worked during the epidemic and followed social distancing guidelines remained antibody‐negative.

**Conclusion:** We conclude that high‐titer CoV‐2 S‐specific antibodies are found in most recovered COVID‐19 patients and in symptomatic contacts, but only rarely in asymptomatic contacts. The assay may help health care providers to monitor disease progression and antibody responses in vaccination trials, to identify health care personnel that likely are resistant to re‐infection, and recovered individuals with high antibody titers that may be suitable asplasma and/or antibody donors.

## Graphical abstract

We report a low‐cost FACS assay for antibodies against the native severe acute respiratory syndrome coronavirus 2 spike protein that does not require recombinant proteins or infectious virus. We demonstrate the utility of the assay by assessing antibody responses of hospitalized patients, health care personnel, and house‐holds of coronavirus diasease 2019 + individuals and their co‐quarantined room mates in the city state of Hamburg

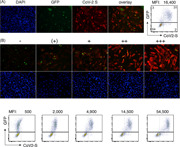

## 1 INTRODUCTION

**Figure 1:**
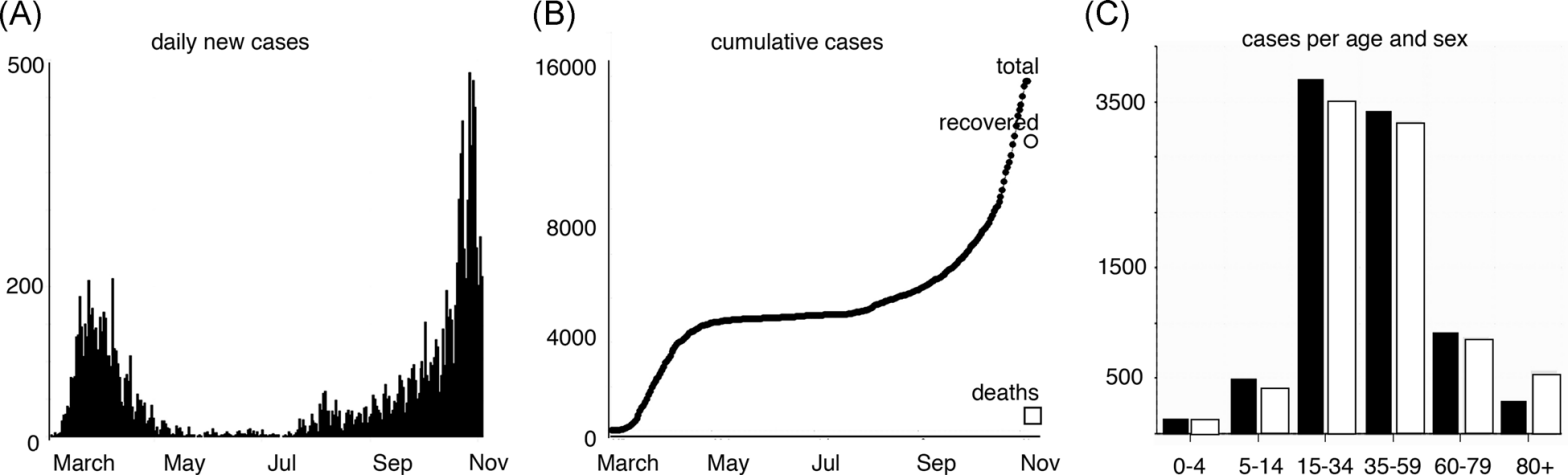
Summary of the coronavirus disease 2019 (COVID‐19) epidemic in the city of Hamburg. The first COVID‐19 cases in Hamburg were reported in early March, predominantly in families returning from traditional skiing vacation in Italy and Austria. (A) Daily new cases between February 23rd and October 22nd. (B) Cumulative cases. As of October 22nd, the total number of COVID‐19 cases reported for Hamburg is 10,407, with 282 deaths and approximately 8000 recoveries. (C) Number of cases per age and sex (male black, female white). Data was obtained from the Robert Koch Institute^2^

Hamburg is a city state of approximately 1.9 Mio inhabitants in Northern Germany. The coronavirus disease 2019 (COVID‐19) epidemic started in Hamburg in early March when families returned from their traditional skiing vacation^1^ and peaked in the middle of April with approximately 220 new cases per day. After having subsided in the summer months to less than five new cases per day, the currently resurging second wave of the epidemic has reached higher levels than during the first wave (>500 new cases per day) (Figure 1). The accumulated total number of cases in Hamburg is approximately 16,000 (880 cases/100,000 inhabitants) with approximately 300 deaths (3% of cases) and 10,000 recovered patients (November 6th, 2020).^2^

Individuals in Hamburg that are tested severe acute respiratory syndrome coronavirus 2 (SARS‐CoV‐2) positive by polymerase chain reaction (PCR)‐assay for coronavirus 2 (CoV‐2) genomic RNA in throat swabs are referred to quarantine in their homes for 14 days together with other members of their household. Patients that develop severe disease are admitted to the University Medical Center Hamburg‐Eppendorf or to another local hospital. Patients that develop respiratory failure are transferred to intensive care and those who go on to develop acute respiratory distress syndrome receive extracorporeal membrane oxygenation. Hamburg has had a surplus of intensive care unit‐beds and respirators throughout the epidemic. Recovered patients and their household contacts are released from quarantine 2 days after recovery from symptoms. The lockdown in Germany is controlled in a step‐wise fashion aiming to keep the number of new daily cases below 50/100,000 inhabitants. Recent analyses indicate that the degree of immunity in the general population is still below 2%.^3^

Several serological assays have been developed to detect CoV‐2‐specific antibodies.^4^, ^5^, ^6^, ^7^ Most of these are based on enzyme‐linked immunosorbent assays (ELISAs) that utilize recombinant proteins, e.g. the receptor‐binding domain (RBD) of the spike protein (S). While the epidemic seems to be under control in China, it is resurging in most European countries and is still spreading rapidly in other regions of the world.^8^ Therefore, there is still an urgent need for serological tests with high specificity and lower costs.

Our lab has expertise in raising antibodies against native membrane proteins by complementary DNA (cDNA) immunization.^9^, ^10^ We routinely use transiently transfected CHO or HEK293T cells to monitor specific antibody responses in immunized animals.^11^ Here we set out to apply this assay for detecting antibodies directed against the native spike protein of SARS‐CoV‐2 in human serum samples. The results demonstrate that this simple low‐cost assay allows specific detection of CoV‐2 S‐specific antibodies. The assay can easily be set up in any research lab equipped with a cell culture facility and a fluorescent microscope or flow cytometer. The required plasmids and cells can be obtained freely from our lab.

## 2 RESULTS

### 2.1 Cotransfection of HEK or CHO cells with plasmids encoding the SARS‐CoV‐2 spike protein and nuclear GFP provides a simple tool to detect antibodies that recognize the spike protein in native conformation

**Figure 2:**
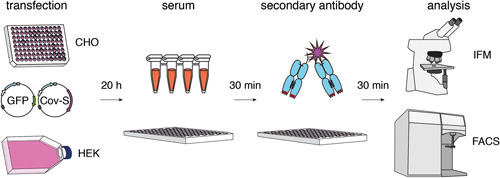
Schematic diagram of the assay. CHO cells are seeded onto a 96‐well culture plate, HEK293 cells onto a tissue culture flask or petri dish. Cells are cotransfected with expression vectors for native full length CoV‐2 S and nuclear GFP. Thirty‐six hours after transfection, CHO cells are fixed for 10 min at RT on the plate with 2% PFA, 20 h after transfection HEK cells are harvested and transferred (non‐fixed) to a 96‐well plate. Cells are incubated for 30 min at RT with human serum samples. Cells are washed and bound antibodies are detected with PE‐conjugated secondary antibodies. CHO cells are analyzed on the 96‐well tissue culture plate by fluorescence microscopy with an inverse microscope equipped with a digital camera. HEK cells are analyzed by flow cytometry. CoV‐2, coronavirus 2; PFA, paraformaldehyde; RT, room temperature

To detect antibodies that recognize the spike protein in native conformation we transfected HEK or CHO cells with a full‐length cDNA expression plasmid followed by immunofluorescence microscopy (IFM) or flow cytometry (Figure 2). Cotransfection with a cDNA expression plasmid encoding green fluorescent protein (GFP) fused to a nuclear localization signal permits distinction of cotransfected cells (green‐fluorescent nuclei) and untransfected cells. The latter serve as negative controls that permit detection of antibodies directed against irrelevant cellular proteins. Cell‐bound CoV‐2 S‐specific antibodies can be detected with a fluorochrome‐conjugated secondary antibody. We use a 96‐well format to simultaneously analyze many samples at a cost of less than 20 € per plate (see Table S1).

**Figure 3:**
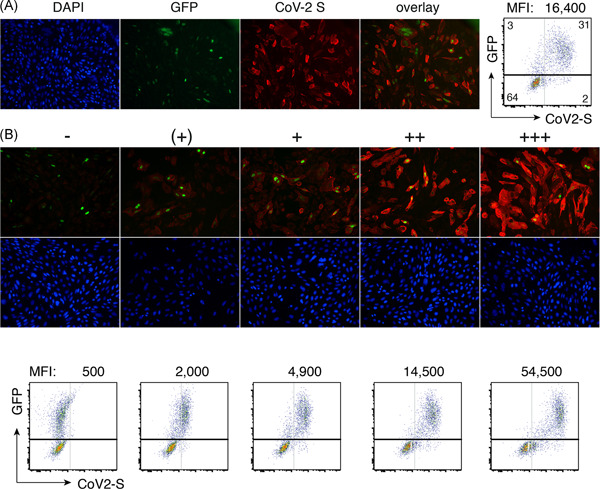
Detection of SARS‐CoV‐2 S‐specific antibodies by immunofluorescence microscopy and flow cytometry. (A) CHO cells or HEK293T cells were cotransfected with cDNA expression constructs for full length CoV‐2 S and nuclear GFP. Thirt‐six hours after transfection, cells were incubated for 30 min with VHH‐72‐hIgG1 heavy chain antibody (hcAb). Cells were washed and bound antibodies were detected with PE‐conjugated secondary antibodies using immunofluorescence microscopy ×20 or flow cytometry. Nuclear DNA was counterstained with DAPI. Numbers in the FACS plot indicate the percentage of cells in the respective quadrants. MFI on top of the FACS plot indicates the CoV‐2 S mean fluorescence intensity of GFP+ cells, that is, of cells in the upper two quadrants. (B) CHO cells (top) and HEK cells (bottom) were transfected and analyzed as in A, except that human serum samples were used instead of recombinant hcAb. The relative staining intensity of CoV‐2 S (red fluorescence) is indicated by −, (+), +, ++, +++. Comparison of fluorescent versus DAPI staining indicates that approximately 20%–40% of cells are cotransfected by nuclear GFP and membrane CoV‐2 S. MFI on top of the FACS plots indicates the mean CoV‐2 S fluorescence intensity of GFP+ cells, that is, of cells in the upper two quadrants. Data are from a single experiment representative of three independent experiments. FACS, fluorescence‐activated cell sorting; cDNA,complementary DNA; CoV‐2, coronavirus 2; DAPI, 4′,6‐diamidino‐2‐phenylindole; MFI, median fluorescent intensity; PE, phycoerythrin; SARS‐CoV‐2, severe acute respiratory syndrome coronavirus 2

To validate the assay, we used the CoV‐S‐specific nanobody VHH‐72^12^ in a human immunoglobulin 1 (IgG1) heavy chain antibody (hcAb) format^13^ (Figure 3). Bound hcAb was detected with PE‐conjugated anti‐human IgG (H + L). The results show that this antibody stains the vast majority of GFP‐transfected cells but not GFP‐negative cells (Figure 3A). The results indicate greater than 90% cotransfection of cells with the expression plasmids for GFP and CoV‐2 S. The expression construct for nuclear GFP can be substituted with constructs for other suitable fluorescent proteins, for example, mitoDSRED or nuclear BFP, the secondary antibodies with other fluorescently labeled secondary antibodies, for example, fluorescein isothiocyanate or Alexa Fluor 647‐conjugates (not shown).

### 2.2 The vast majority of COVID‐19 patients develop moderate to very high levels of CoV‐2 S‐specific antibodies

We next set out to monitor the antibody responses of SARS‐CoV‐2‐infected individuals that had been hospitalized at the University Medical Center in Hamburg. We also analyzed serum samples from members of our lab that had continued to work during the epidemic while following the social distancing recommendations (lab work is organized in two shifts with coworkers wearing masks and maintaining a distance of >1.5 m). Sera were treated for 30 min at 56°C to inactivate complement components and SARS‐CoV‐2 virions. Figure 3B shows representative microscopy and flow cytometry results. The staining intensities allow a semiquantitative assessment of antibody levels by IFM (indicated by −, +, ++, +++) and a quantitative assessment by flow cytometry (indicated by the mean fluorescence intensity of GFP + cells). The results of IFM and fluorescence‐activated cell sorting (FACS) analyses of representative 96‐well plates are shown in Supplementary Figures 1 and 2, respectively. Cells were cotransfected with expression vectors for CoV‐S and GFP at a 10:1 molar ratio, which could explain cells expressing CoV‐S but little if any GFP.

Table 1 summarizes the results of the samples from hospitalized patients, Table S1 the results of samples from healthy coworkers. The results obtained by immunofluorescence microscopy and flow cytometry concord very well. Antibodies became detectable in all samples from hospitalized patients with 8–14 days after disease onset (Table 1). There is a slight tendency toward higher antibody levels in patients with more severe clinical symptoms. In contrast, all samples of healthy coworkers did not show any detectable antibodies (Table S1). We further analyzed 221 pre‐COVID‐19 samples with our FACS assay (Figure S3).

**Table 1:** Summary of antibody levels for hospitalized COVID‐19 patients at the University Medical Center Hamburg

**Patient**	**Age**	**Days after disease onset**	**Disease severity**	**IFM**	**FACS**	**Plate**
1	73	14	+++	−	1.5	1D09
1	73	42	−	+++	73.1	2F04
2	58	13	+++	+	1.0	1E09
3	52	6	++	−	0.6	1E08
4	85	5	+++	−	1.0	1D08
4	85	12	+++	(+)	8.0	1A08
4	85	41	−	+++	56.2	2G04
5	60	9	+++	−	0.9	1G09
6	57	10	++	(+)	3.8	1C09
7	38	20	++	++	23.1	1A12
8	50	8	++	+	0.9	1F09
9	63	11	+++	++	5.5	1B10
10	77	6	+++	u	1.5	1H09
11	75	10	++	(+)	6.2	1C10
12	29	7	++	++	48.0	1A09
13	60	5	+++	(+)	2.0	1B09
13	60	12	+++	++	8.1	1F11
14	34	10	++	(+)	4.6	2H09
14	34	17	−	+	20.0	2F01
14	34	36	−	++	54.4	2D04
15	37	5	++	−	1.1	1A10
15	37	12	++	++	4.9	1B12
16	61	4	++	−	0.6	1D10
17	73	9	+++	(+)	8.9	2H01
18	59	15	++	+++	54.5	2E02
19	57	10	+++	++	35.4	2E01
19	57	16	+++	+++	65.2	2G03
20	38	4	++	−	1.0	1A11
21	27	10	++	+	9.2	2D01
21	27	28	−	++	43.3	2A04
22	42	8	++	+	2.9	2B02
22	42	16	++	+++	69.0	2H04
23	60	7	++	+	11.8	2C02
23	60	21	−	+	11.7	2B04
23	60	31	−	++	9.1	2D06
24	47	4	+	u	1.2	2G02
24	47	8	+	u	2.7	2F03
24	47	21	−	u	9.5	2C04
24	47	29	−	u	8.3	2B05
25	65	3	+++	−	0.9	2F02
26	78	0	+	−	3.4	2H05
26	78	14	++	+++	67.1	2F07

*Note*: Data are from one experiment representative of two independent experiments.

Abbreviations: COVID‐19, coronavirus disease 2019; FACS, fluorescence‐activated cell sorting; IFM, immunofluorescence microscopy.

**Figure 4:**
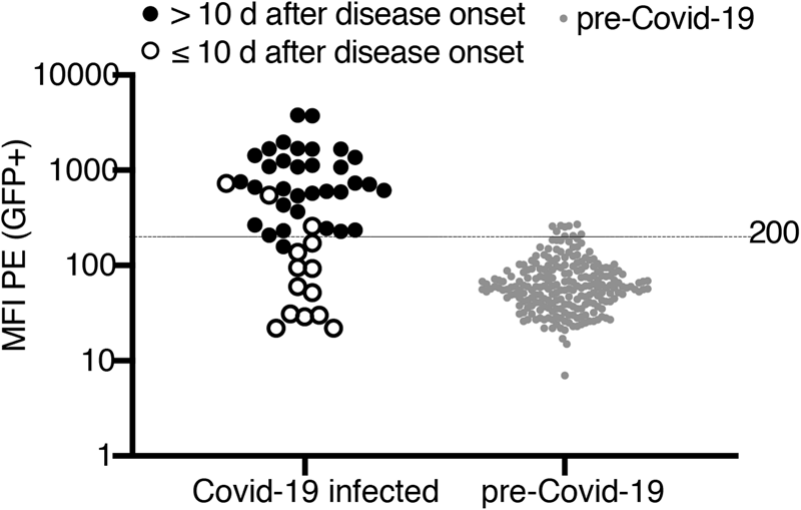
Threshold, sensitivity and specificity of the FACS assay. (A) Antibody levels in 50 samples of COVID‐19 infected individuals and 221 samples of pre‐COVID‐19 individuals were determined by flow cytometry using native spike protein expressed on the cell surface of CoV‐2‐S/GFP‐cotransfected HEK cells. Samples obtained from COVID‐19 patients within the first 10 days after disease onset are indicated by open circles, samples obtained from the same patients more than 10 days after disease onset are indicated by closed black circles. The threshold was set at the MFI of 200 (indicated by the long dashed line). (B) Specificity and sensitivity of the assay were calculated using samples obtained from COVID‐19 patients more than 10 days after disease onset and all samples from individuals before December 2019. Data shown are from a single experiment and are representative of two independent experiments. COVID‐19, coronavirus disease 2019; FACS, fluorescence‐activated cell sorting; MFI, mean fluorescence intensity; SARS‐CoV‐2, severe acute respiratory syndrome coronavirus 2

We used the reactivities of pre‐COVID‐19 samples to set a threshold (Figure 4). The results show that nine of 221 tested pre‐COVID‐19 samples were false positive, that is, had values slightly above the threshold. Conversely, one of 33 samples from eight COVID‐19 patients that were obtained more than 10 days after disease onset was false negative, that is, had a value slightly below the threshold. This corresponds to a calculated sensitivity of 97% and a specificity of 96%. Of the 14 samples from eight COVID‐19 patients obtained less than 10 days after disease onset, three samples showed values above and eleven samples showed values below the threshold. The low levels of antibodies that bind the native spike protein of CoV‐2 in some pre‐COVID‐19 samples may represent cross‐reactive antibodies induced by a prior coronavirus infection.

### 2.3 Eight of nineteen co‐quarantined close contacts of COVID‐19 patients did not develop any detectable CoV‐2 S‐specific antibodies

**Figure 5:**
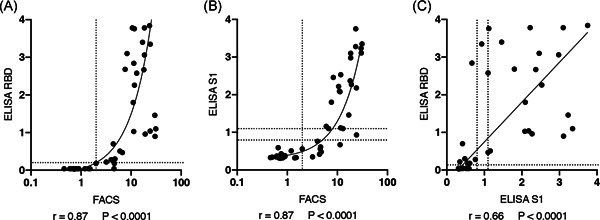
Correlation of CoV‐2 S‐specific antibody levels in household patients assessed by FACS and ELISA. Antibody levels in 60 samples of 27 individuals (18 COVID‐19‐infected individuals and 9 co‐quarantined contacts, Table 2) were determined by flow cytometry (native spike protein expressed on the cell surface of transfected HEK cells), Euroimmune ELISA (recombinant S1 domain), or the Wantai ELISA (recombinant RBD). Pearson correlation coefficients and p values (two‐tailed) were determined using prism 8.4.3 (Graphpad). Interpolation was performed by robust line regression using prism 8.4.3. Dashed lines indicate the thresholds‐borderline‐spans for the respective assays. Data shown are from a single experiment and are representative of two independent experiments performed with the 60 samples of 27 individuals. COVID‐19, coronavirus disease 2019; ELISA, enzyme‐linked immunosorbent assay; FACS, fluorescence‐activated cell sorting

We next analyzed 60 samples from 27 individuals of eight households in which at least one member had contracted COVID‐19 and had been co‐quarantined with his/her family members or household contacts (Table 2, for asymptomatic household contacts, numbers in parentheses refer to the days after disease onset of their COVID‐19+ household contacts. Further details on these households are provided in the supplementary materials). We also analyzed these 60 samples with two well‐established ELISAs (Table 2). The results show very good concordance of our FACS assay with those of the Wantai and Euroimmune assays (Figure 5). Differences in FACS versus ELISA signal intensities observed for some samples likely are due to the different antigens employed in these assays. While our assay uses native, glycosylated spike protein displayed on the cell surface of living cells, the Wantai and Euroimmune employ subdomains, that is, the RBD domain and the S1 domain (the N‐terminal and RBD domains) as purified recombinant proteins, respectively.

**Table 2:** Summary of antibody levels of quarantined COVID‐19+ symptomatic patients and their co‐quarantined K1‐close contacts

**House hold**	**Age**	**Days after disease onset**	**Disease severity**	**IFM native S**	**FACS native S**	**ELISA S1**	**ELISA RBD**	**Plate**
H1‐1	64	7	++	−	0.5	0.34	n.d.	2A07
H1‐1	64	10	+	+	4.4	0.42	0.70	2H08
H1‐1	64	16	++	+	19.1	2.27	0.96	1F08
H1‐1	64	22	−	++	29.8	3.23	1.46	1C08
H1‐1	64	31	−	++	31.1	3.35	1.10	1H12
H1‐1	64	42	−	+++	30.1	3.11	0.90	2E08
H1‐2	62	4	−	−	0.8	0.40	0.04	2B07
H1‐2	62	16	−	+	16.9	1.46	3.40	1B08
H1‐2	62	25	−	+	18.0	2.37	2.67	1G12
H1‐2	62	31	−	++	23.2	3.28	n.d.	2B08
H1‐2	62	36	−	++	24.1	0.93	3.35	2H03
H2‐1	41	61	−	++	8.3	2.46	3.10	2G07
H2‐2	35	57	−	++	11.0	2.09	1.80	2H07
H3‐1	50	24	−	++	23.3	3.75	3.84	1B05
H3‐2	56	14	−	+	11.6	1.12	3.76	1A05
H3‐3	21	12	−	+	10.6	2.22	3.78	1H06
H3‐4	19	(24)	−	++	7.6	1.80	2.68	1G06
H4‐1	29	50	−	+	11.7	2.53	2.26	2A05
H4‐2	26	45	−	−	1.2	0.51	0.04	2H06
H4‐3	30	44	−	+	11.3	2.08	1.03	2G06
H4‐4	25	41	−	++	18.5	2.98	3.06	2F06
H4‐5	26	32	−	+	6.7	1.10	0.47	2E06
H5‐1	54	65	−	++	24.0	2.18	1.04	2H11
H5‐2	4	(65)	−	u	0.9	0.38	0.04	2G11
H6‐1	66	56	−	+	18.5	3.10	3.78	2A11
H6‐2	60	56	−	+	6.0	1.16	0.51	2H12
H7‐1	37	4	+	−	0.8	0.43	0.04	1D11
H7‐1	37	7	+	−	2.0	0.56	0.18	1E12
H7‐1	37	11	−	u	4.8	0.48	0.30	2D09
H7‐1	37	17	−	u	4.6	0.61	0.18	2C03
H7‐1	37	22	−	−	4.0	0.76	0.28	2F05
H7‐1	37	37	−	u	4.6	0.56	0.04	2C09
H7‐2	34	−2	−	−	0.7	0.32	0.03	1E11
H7‐2	34	1	+	−	0.7	0.59	0.04	1F12
H7‐2	34	5	+	+	3.2	0.35	0.22	2C01
H7‐2	34	11	+	+	11.3	0.67	2.84	2D03
H7‐2	34	26	−	++	12.9	1.10	2.58	2G05
H7‐3	32	(7)	−	−	0.8	0.33	0.04	1B11
H7‐3	32	(11)	−	−	0.7	0.31	0.04	1C12
H7‐3	32	(17)	−	−	1.5	0.33	0.04	2H02
H7‐3	32	(22)	−	−	0.9	0.36	0.04	2A03
H7‐3	32	(37)	−	−	0.8	0.32	0.04	2D05
H7‐4	29	(7)	−	−	0.5	0.33	0.04	1C11
H7‐4	29	(11)	−	−	0.7	0.32	0.04	1D12
H7‐4	29	(17)	−	−	1.4	0.32	0.04	2A01
H7‐4	29	(22)	−	−	0.6	0.37	0.04	2B03
H7‐4	29	(37)	−	−	0.6	0.35	0.04	2E05
H8‐1	29	9	−	++	9.5	0.41	0.28	1F02
H8‐1	29	20	−	++	10.3	0.60	0.31	1H03
H8‐1	29	40	−	+	5.6	0.47	0.06	2E11
H8‐2	30	(9)	−	−	0.8	0.63	0.04	1E02
H8‐2	30	(20)	−	−	0.9	0.67	0.04	1G03
H8‐3	31	(9)	−	−	0.7	0.32	0.03	1D02
H8‐3	31	(11)	−	−	0.8	0.32	0.04	1F03
H8‐4	26	(9)	−	−	1.2	0.48	0.03	1C02
H8‐4	26	(20)	−	−	1.1	0.42	0.04	1E02
H8‐5	27	(9)	−	−	1.0	0.36	0.04	1B02
H8‐5	27	(20)	−	−	1.2	0.36	0.04	1D03
H8‐6	31	(9)	−	−	0.9	0.40	0.03	1A02
H8‐6	31	(20)	−	−	1.1	0.45	0.04	1C03

*Note*: Data are from one experiment representative of two independent experiments.

Abbreviations: COVID‐19, coronavirus disease 2019; ELISA, enzyme‐linked immunosorbent assay; FACS, fluorescence‐activated cell sorting; IFM, immunofluorescence microscopy.

The eight members of households H1, H2, and H3 all reported symptoms and developed antibodies with one exception: H3‐4 (19‐year) did not recall any symptoms beyond a mild sore throat for 1 day and was repeatedly tested SARS‐CoV‐2 negative by PCR. He did develop CoV‐2 S‐specific antibodies, suggesting that he may have been the first member of his family to acquire SARS‐CoV‐2, but had already eliminated the virus by the time of his PCR‐tests. In households H4‐H8, five members that reported symptoms also developed antibodies; one member (H4‐2, 26‐year) reported mild symptoms, was not tested by PCR and did not develop any detectable CoV‐2 specific antibodies. The other eight members of H4‐H8 did not show symptoms and did not develop any detectable antibodies.

### 2.4 Serial samples indicate maintenance of plateau antibody levels for up to 120 days

**Figure 6:**
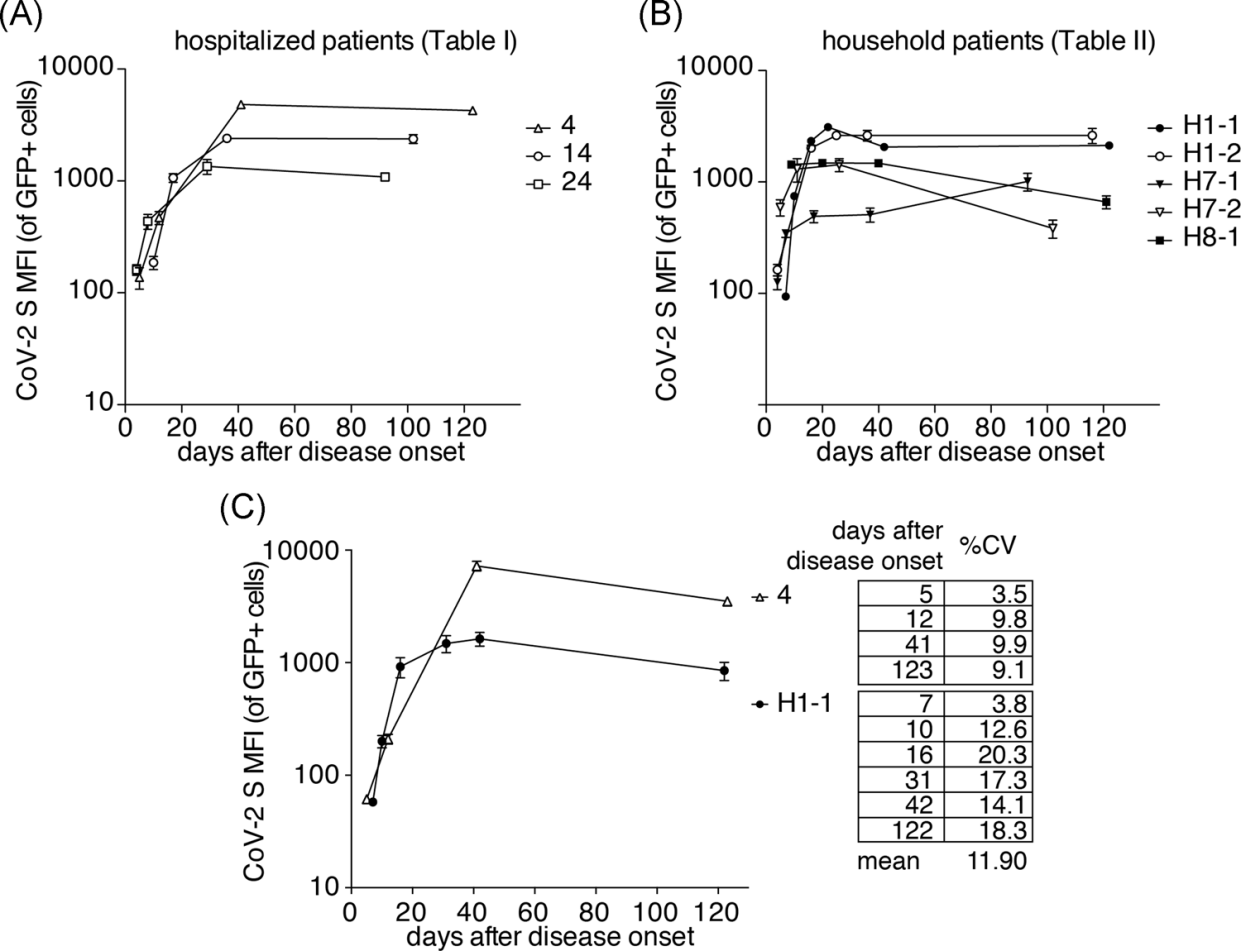
Antibody dynamics in eight patients. HEK293T cells were cotransfected with cDNA expression constructs for full length CoV‐2 S and nuclear GFP as in Figure 3. CoV‐2 S‐specific antibody levels in (A) hospitalized patients and (B) household patients were assessed by flow cytometry in triplicate samples. Numbers refer to the patient assignments in (A) Tables 1 and 2, respectively. Error bars indicate standard deviation of three technical replicates. Data are from a single experiment that is representative of three independent experiments performed. (C) The ten samples of (A) patients 4 and (B) H1‐1 were re‐analyzed in five independent experiments. The results were used to calculate the average coefficient of variance between duplicates (% CV). cDNA, complementary DNA; CoV‐2, coronavirus 2; CV, coefficient of variation; MFI, mean fluorescence intensity

Serial samples were available from three SARS‐CoV‐2‐infected hospitalized patients and from five SARS‐CoV‐2‐infected household patients. We analyzed these samples in parallel in triplicates with our FACS assay using the mean fluorescence intensity as an indicator of the levels of antibodies against the native spike protein of SARS‐CoV‐2 (Figure 6). The results show a rapid rise in antibody levels within the first two weeks after disease onset. Antibody levels typically reach a plateau within 20–40 days. Most patients maintained a plateau antibody level for the duration of analysis (up to 120 days after disease onset). To assess the reproducibility of the assay, we performed independent repeat measurements of 10 samples covering the whole spectrum of reactivities from hospitalized patient H4 and household patient H 1‐1 (Figure 6C). The results confirm a very good reproducibility with a calculated % coefficient of variation from 3.5 to 20.3 (mean 11.9).

## 3 DISCUSSION

We report a simple, low‐cost assay for detecting antibodies against the native SARS‐CoV‐2 spike protein. The assay may help health care providers to monitor disease progression and antibody responses in vaccination trials, to identify health care personnel that likely are resistant to re‐infection, and recovered individuals with high antibody titers that may be suitable as plasma and/or antibody donors.^7^, ^14^, ^15^, ^16^

Low costs, simple set up, and incorporated controls to detect false positives, are the main advantages of our assay over established ELISA and peptide‐based assays. Moreover, ELISA assays depend on the costly production and purification of recombinant proteins. Peptide arrays often miss antibodies directed against conformational epitopes. Use of purified plasmid DNAs and natively expressed spike protein likely account for the high specificity and reproducibility of our FACS assay. Cotransfection of cells with a cDNA encoding a fluorescent protein allows the clear distinction of “false positives” caused by antibodies that recognize irrelevant antigens. However, the FACS assay requires more time, equipment and operator skills than commercial ELISAs. It may thus more suitable for applications in research labs rather than for clinical diagnostic labs.

Our results with samples from hospitalized and household patients and their close contacts revealed that all individuals that had recovered from a symptomatic COVID‐19 infection developed moderate to high titers of CoV‐2 S‐specific antibodies. Typically, antibodies became detectable 6–12 days after disease onset and reached a plateau level within three weeks. None of the analyzed laboratory personnel of the University Medical Center showed any detectable SARS‐CoV‐2 S‐specific antibodies. Remarkably, eight of nineteen analyzed close contacts of COVID‐19 patients in household quarantine did not develop any symptoms and also did not develop any detectable CoV‐2 S‐specific antibodies. These results indicate that these close contacts were not infected and consequently did not develop antibody‐mediated immunity even during co‐quarantine with a symptomatic patient. This conclusion is in line with reports from other regional outbreaks of COVID‐19.^4^, ^6^, ^17^, ^18^

We conclude that individuals that have recovered from COVID‐19 carry moderate to high titers of antibodies directed against the native CoV‐2 spike protein. In contrast, many asymptomatic close contacts are antibody‐negative. These individuals presumably continue to be susceptible to COVID‐19 infection or might have a T‐cell driven immunity from previous coronavirus infections.^19^

## 4 MATERIALS AND METHODS

### 4.1 Serum samples

Serum or plasma was obtained from patients and healthy volunteers after informed consent. For presentation in the tables, clinical symptoms were graded as follows: − none; (+) mild (fever, weakness); + moderate (headache, high fever, anosmia); ++ severe (requiring hospitalization); +++ life threatening (intensive care and respiration aid). Further clinical information is provided as supplementary material to Table 2. Serum samples were incubated for 30 min at 56°C to inactivate complement and coronaviruses^20^ before use. All results shown in this paper were obtained with serum samples at a dilution of 1:100−1:200.

### 4.2 Expression constructs

The codon‐optimized intact open reading frame of the native spike protein of SARS‐CoV‐2 (GenBank MN908947) was produced by gene synthesis (pTwistCoV2S). A similar construct (NR‐52310) was kindly provided by the Krammer lab in New York.^4^ The expression construct for nuclear GFP (pCDNA6‐GFPnls) was cloned in our lab as described previously^11^ by fusion of the coding sequences of GFP upstream of the DNA‐binding domain of the transcription factor LKLF. Plasmids were purified with standard mini or maxi prep kits (Qiagen). These plasmids are available upon request from our lab.

### 4.3 Recombinant CoV‐S‐specific heavy chain antibody

The codon‐optimized open reading frame of the SARS RBD‐specific nanobody VHH‐72^12^ flanked by NcoI and NotI restriction enzyme sites was generated by gene synthesis and cloned into the pCSE2.5 vector^21^ upstream of the hinge, CH2 and CH3 domains of human IgG1.^22^ VHH‐72‐human IgG1 hcAbs were generated by transient transfection of HEK‐6E cells^23^ cultivated for 6 days in serum‐free medium. Cells were pelleted by centrifugation and hcAb‐containing supernatants were stored at 4°C until use. Yield of recombinant hcAbs was estimated by sodium dodecyl sulfate‐polyacrylamide gel electrophoresis and Coomassie‐staining using 10 µl culture supernatant/well^9^ to be in the range of 20–200 µg/ml. Supernatants were used at a dilution of 1:20.

### 4.4 Cells and cell transfections

Chinese hamster ovary cells (CHO) and human embryonal kidney cells (HEK293T) were obtained from the DSMZ, Braunschweig. Cells were maintained in Dulbecco's modified Eagle's medium (DMEM) medium supplemented with 10% fetal calf serum, 1% sodium‐pyruvate, 1% non‐essential amino acids and 1% glutamine. Aliquots of these cells are available from our lab. Cells were passaged every 2–3 days by trypsinization (Gibco) and passaged at a dilution of 1:4–1:10. A semi‐confluent 10 cm petri dish or T75 culture flask is sufficient for transfection of a single 96 well plate. HEK cells were transfected in the T75 flask, CHO cells were passaged onto a 96 well plate before transfection. A stock solution of Polyethylimidine (Sigma) was prepared in sterile water (160 µg/ml). 10 µg of the SARS‐CoV‐2 S expression vector, 1 µg of the GFP expression vector, and 20 µg of PEI are sufficient for transfection of a 96‐well plate. Plasmid DNAs were adjusted to a volume of 500 µl with 150 mM NaCl solution. Five hundred micoliters of PEI solution (40 µg/ml in 150 mM NaCl) was added slowly to the DNA, vortexed for 10 s and rested for 20 min. For transfection of HEK cells, the entire DNA solution was pipetted dropwise onto the medium in the T75 flask using a 5 ml pipette; for transfection of CHO cells the DNA solution was distributed onto the 96 wells using a multichannel pipette (10 µl per well). Cells were incubated overnight and examined under an inverse fluorescent microscope for appearance of cells with a green fluorescent nucleus.

### 4.5 Immunofluorescence microscopy

Twenty to 36 h after transfection, CHO cells were gently washed once with warm phosphate‐buffered saline (PBS) and were incubated for 10 min in PBS containing 2% paraformaldehyde. The PBS/paraformaldehyde solution was discarded and replaced with 200 µl PBS containing 0.02% Na‐Azide. Wells were used directly for IFM analyses or stored at 4°C for up to 6 weeks before use (wrap the plate with parafilm to prevent desiccation). Cells were incubated for 60 min at 4°C with serum diluted 1:100 in DMEM containing 10% fetal calf serum (FCS). Cells were washed twice with PBS and incubated further for 20 min at RT with PE‐conjugated anti‐human IgG (H + L) F(ab')2 (Jackson) (diluted 1:200 in DMEM/FCS). Cells were washed twice with PBS and analyzed with an EVOS fluorescence microscope (Thermo Fisher Scientific) equipped with a digital camera.

### 4.6 Flow cytometry

Twenty to 36 h after transfection, HEK cells were resuspended by gentle pipetting in DMEM containing 10% FCS. Cells were washed by centrifugation, resuspended in PBS containing 0.2% BSA and distributed onto a round bottom 96‐well plate (10^5^ cells/well). Cells were incubated for 60 min at 4°C with serum diluted 1:200 in PBS/0.2% BSA, washed twice with PBS/0.2% BSA, and incubated further for 20 min at RT with PE‐conjugated anti‐human IgG (H + L) F(ab')2 (Jackson) (diluted 1:200 in PBS/0.2% BSA). Cells were washed twice with PBS/0.2% BSA and analyzed with a FACS Celesta or a FACS Canto flow cytometer (BD Biosciences). Scores for IFM and FACS in Tables 1 and 2 were determined as described in Figure 3 (FACS MFI values were divided by 1000 for better legibility). Plate coordinates in Tables I and II correspond to those in Figures S2 and S3.

### 4.7 ELISA

The EUROIMMUNE ELISA and Wantai ELISA were performed according to the manufacturers' instructions. For the EUROIMMUNE ELISA, data is presented as a ratio of the optical densoity (OD) of the sample divided by the OD of an internal control (Calibrator) (ratio > 1.1 = “positive,” 0.8 − 1.1 = “borderline”, <0.8 = “negative”). The Wantai ELISA data is presented as a ratio of the sample OD divided by the “cut‐off.” The cut‐off is set to 0.19 if the average of the negative controls is below 0.03, which was the case in our assay.

### 4.8 Statistics

Pearson correlation coefficients, *p* values (two‐tailed) and coefficient of variation were determined using prism 8.4.3 (Graphpad). Interpolation was performed using line settings and robust regression using prism 8.4.3 (Graphpad). Triplicates were measured and plotted as mean ± *SD* using error bars on both sides.

### 4.9 Study approval

The procedure followed in the study were approved by the local ethics committee (Ethik‐Kommission der Ärztekammer Hamburg PV4780 and PV5139 for samples from healthy donors, and PV7298 for samples from COVID‐19 patients). Written informed consent was obtained from recruited patients and healthy individuals.

## CONFLICT OF INTERESTS

All authors declare that there are no conflict of interests.

## AUTHOR CONTRIBUTIONS

Stephan Menzel and Friedrich Koch‐Nolte contributed to completion of conceptualization and project administration. Tobias Stähler, Julia Hambach, Stephan Menzel, Thomas Eden, Dorte Wendt, and Natalie Tode contributed to investigation. Tobias Stähler, Julia Hambach, Stephan Menzel, Marcus Altfeld, Christine Dahlke, Anahita Fathi, Marylyn M. Addo, and Friedrich Koch‐Nolte contributed to data analysis and data curation. Friedrich Haag, Eva Tolosa, Marcus Altfeld, Christine Dahlke, Anahita Fathi, and Marylyn M. Addo contributed to resources. Friedrich Koch‐Nolte contributed to conpletion of writing. Friedrich Koch‐Nolte, Stephan Menzel, and Marylyn M. Addo contributed to supervision. Friedrich Haag, Eva Tolosa, Marcus Altfeld, Marylyn M. Addo, and Friedrich Koch‐Nolte contributed to funding acquisition. Open Access funding enabled and organized by Projekt DEAL.

## Supporting information

Supporting information.

## Data Availability

Cells and plasmids are available from the Koch‐Nolte‐lab upon request (nolte@uke.de). The data that support the findings of this study are available from the corresponding author upon reasonable request.
