# Supplementary material for: A simple, sensitive, and low‐cost FACS assay for detecting antibodies against the native SARS‐CoV‐2 spike protein
Source: Immun Inflamm Dis. 2021 May 12;9(3):905–17. doi: 10.1002/iid3.446 (PMC8239943; doi:10.1002/iid3.446)
Supplement: Supplementary file 1 — Supporting information. [file IID3-9-905-s001.pdf]

Supplementary Material

A simple, sensitive, low-cost FACS assay for detecting antibodies against the native SARS-CoV-2 spike protein

Julia Hambach[a*], Tobias Stähler[a*], Thomas Eden[a], Dorte Wendt[a], Natalie Tode[a], Friedrich Haag[a], Eva Tolosa[a], Marcus Altfeld[a,b], Anahita Fathi[c,d,e], Christine Dahlke[c,d,e], Marylyn M. Addo[c,d,e], Stephan Menzel[a#], Friedrich Koch-Nolte[a#]

[a] *Institute of Immunology, University Medical Center Hamburg-Eppendorf, Hamburg, Germany;* [b] *Department of Virus Immunology, Heinrich Pette Institute, Leibniz Institute for Experimental Virology, Hamburg, Germany;* [c] *Section Infectious Diseases, I. Medical Clinic and Polyclinic, University Medical Center Hamburg-Eppendorf, Hamburg, Germany;* [d] *Department of Clinical Immunology of Infectious Diseases, Bernhard Nocht Institute for Tropical Medicine, Hamburg, Germany;* [e] *German Center for Infection Research, Partner Site Hamburg-Lübeck-Borstel-Riems, Hamburg, Germany.*

Supplementary Figure 1

IFM analyses of 188 serum samples from two representative 96 well plates. Cells were stained and analyzed as described in Figure 2.

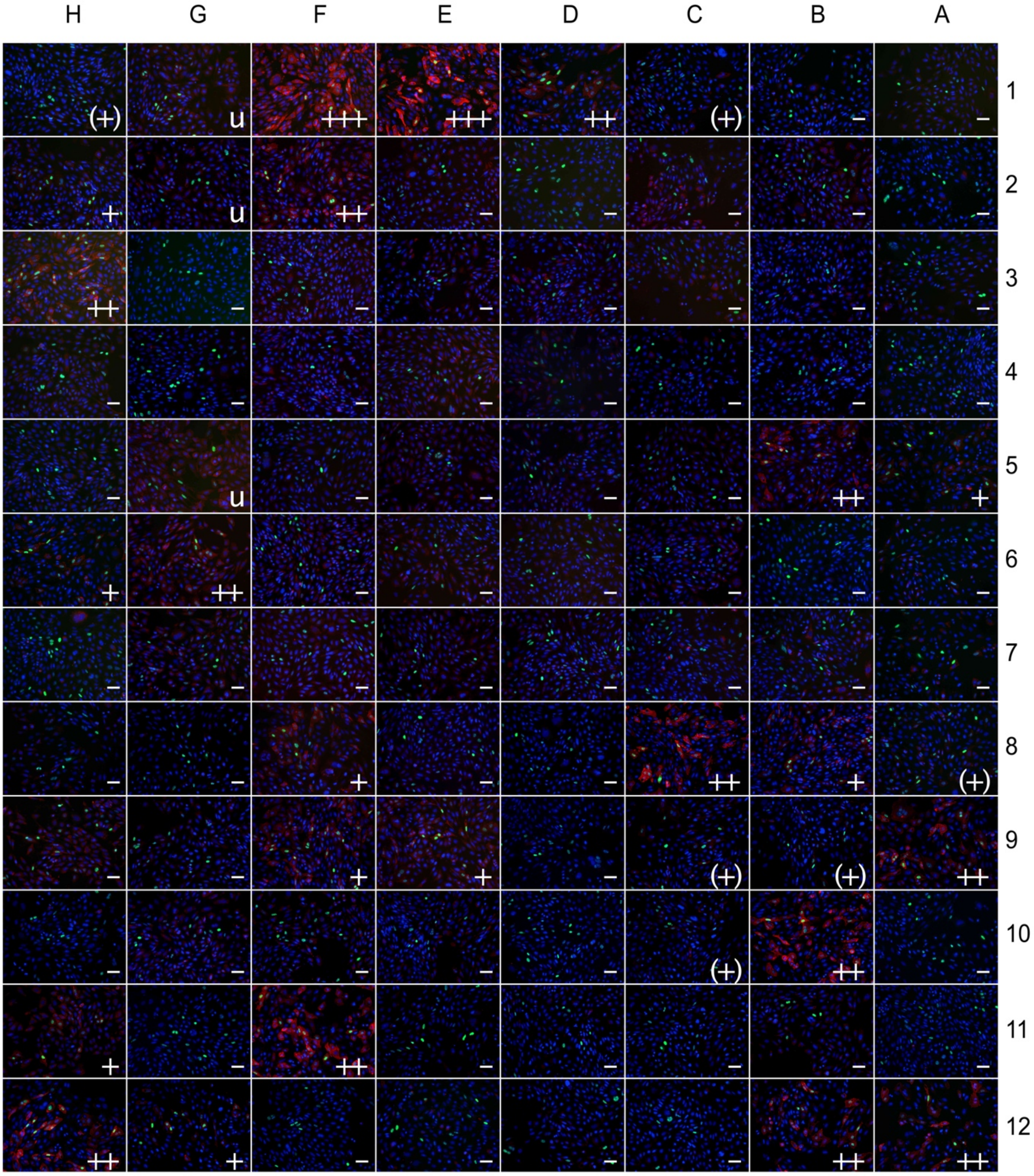

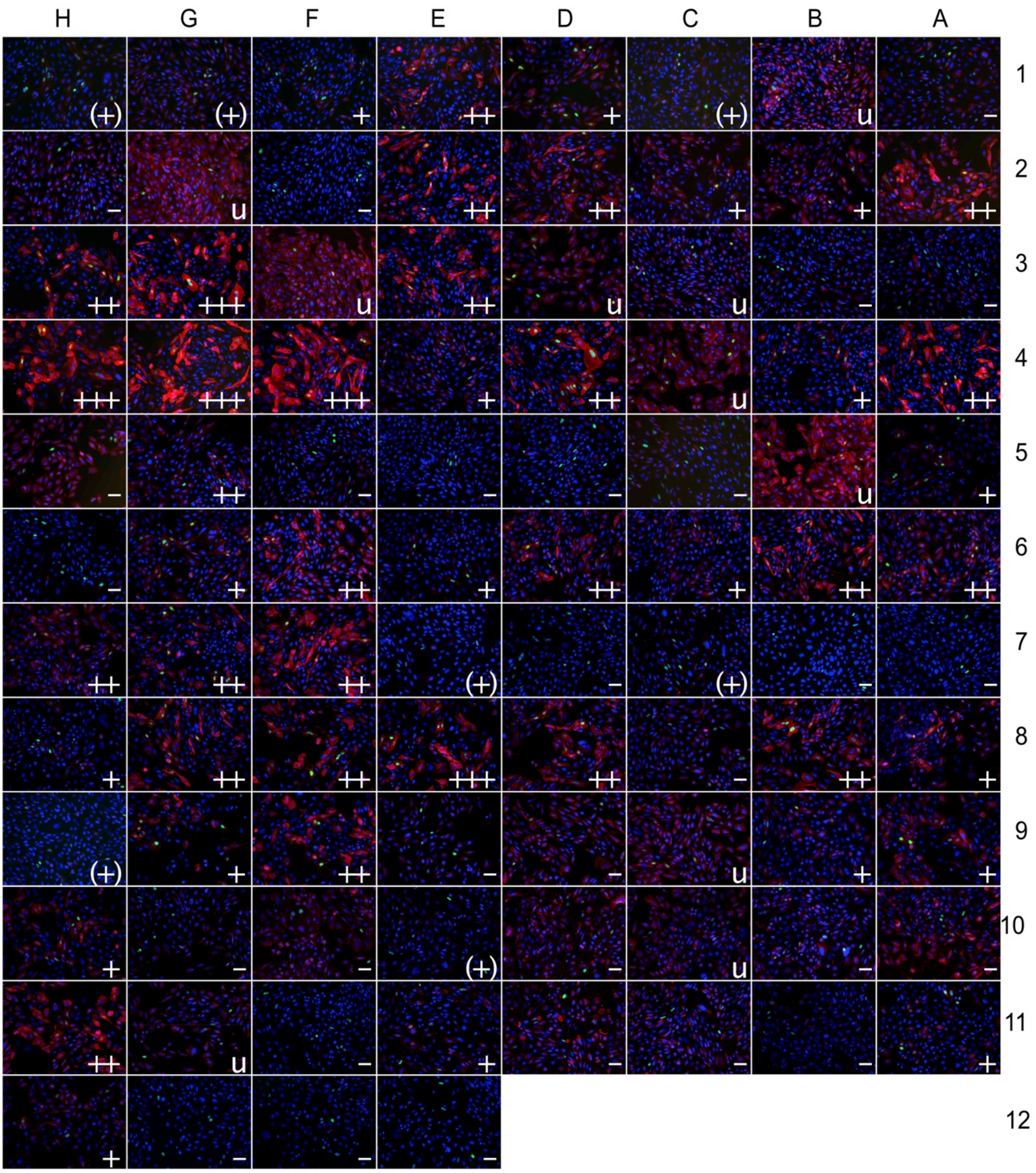
H G F E D C B A
1 (+) (+) + ++ + (+) u −
2 − u − ++ ++ + + ++
3 ++ +++ u ++ u u − −
4 +++ +++ +++ + ++ u + ++
5 − ++ − − − − u +
6 − + ++ + ++ + ++ ++
7 ++ ++ ++ (+) − (+) − −
8 + ++ ++ +++ ++ − ++ +
9 (+) + ++ − − u + +
10 + − − (+) − u − −
11 ++ u − + − − − +
12 + − − −

Supplementary Figure 2:
FACS analyses of 188 serum samples from two representative 96 well plates. Cells were stained and analyzed as described in Figure 2.

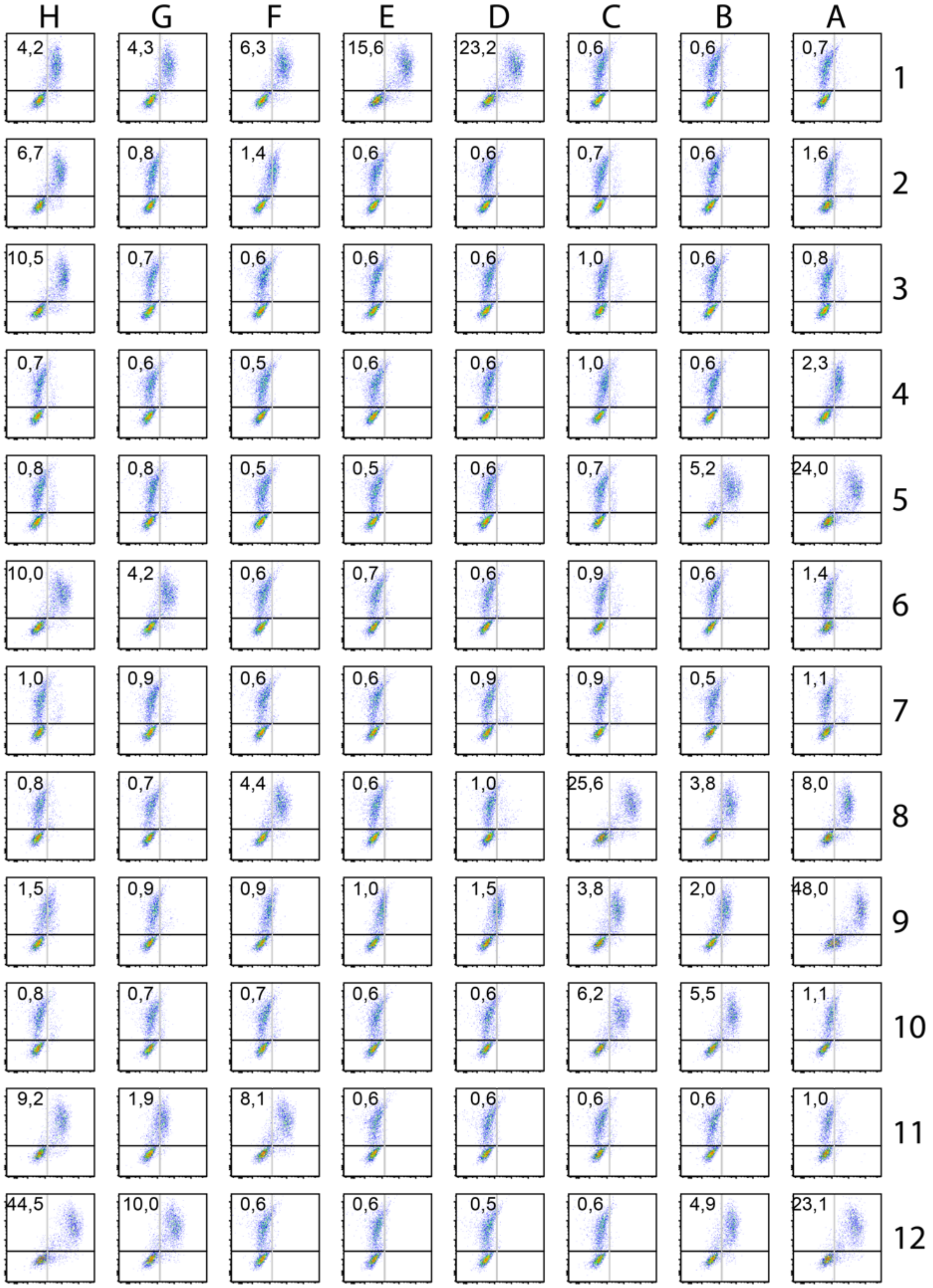

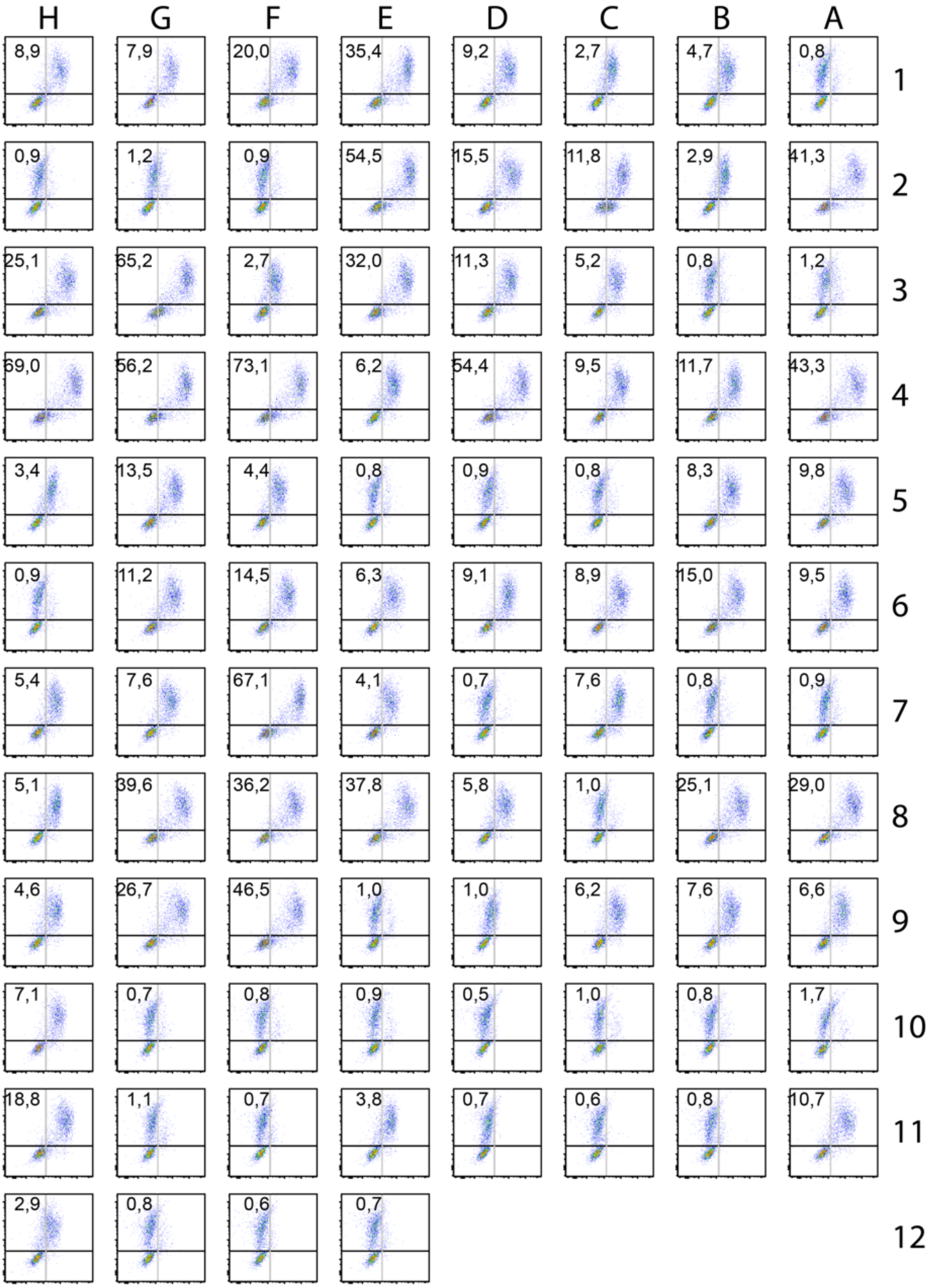
H G F E D C B A
1 8,9 7,9 20,0 35,4 9,2 2,7 4,7 0,8
2 0,9 1,2 0,9 54,5 15,5 11,8 2,9 41,3
3 25,1 65,2 2,7 32,0 11,3 5,2 0,8 1,2
4 69,0 56,2 73,1 6,2 54,4 9,5 11,7 43,3
5 3,4 13,5 4,4 0,8 0,9 0,8 8,3 9,8
6 0,9 11,2 14,5 6,3 9,1 8,9 15,0 9,5
7 5,4 7,6 67,1 4,1 0,7 7,6 0,8 0,9
8 5,1 39,6 36,2 37,8 5,8 1,0 25,1 29,0
9 4,6 26,7 46,5 1,0 1,0 6,2 7,6 6,6
10 7,1 0,7 0,8 0,9 0,5 1,0 0,8 1,7
11 18,8 1,1 0,7 3,8 0,7 0,6 0,8 10,7
12 2,9 0,8 0,6 0,7

Supplementary Figure 3:
FACS analyses of 221 pre Covid-19 serum samples. Cells were stained and analyzed as described in Figure 3. Samples H1, G1, and F1 are control samples from Covid 19 patients

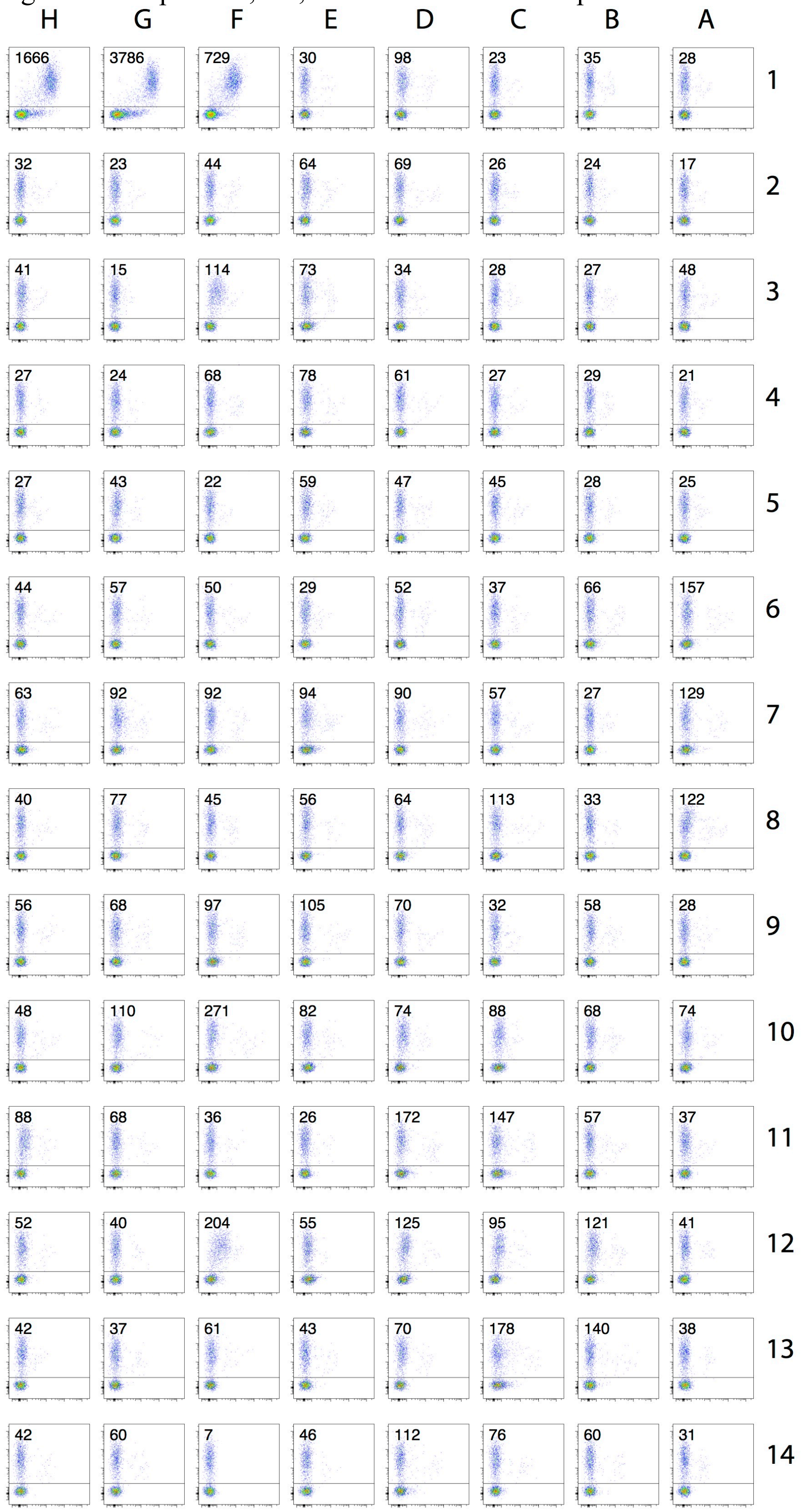

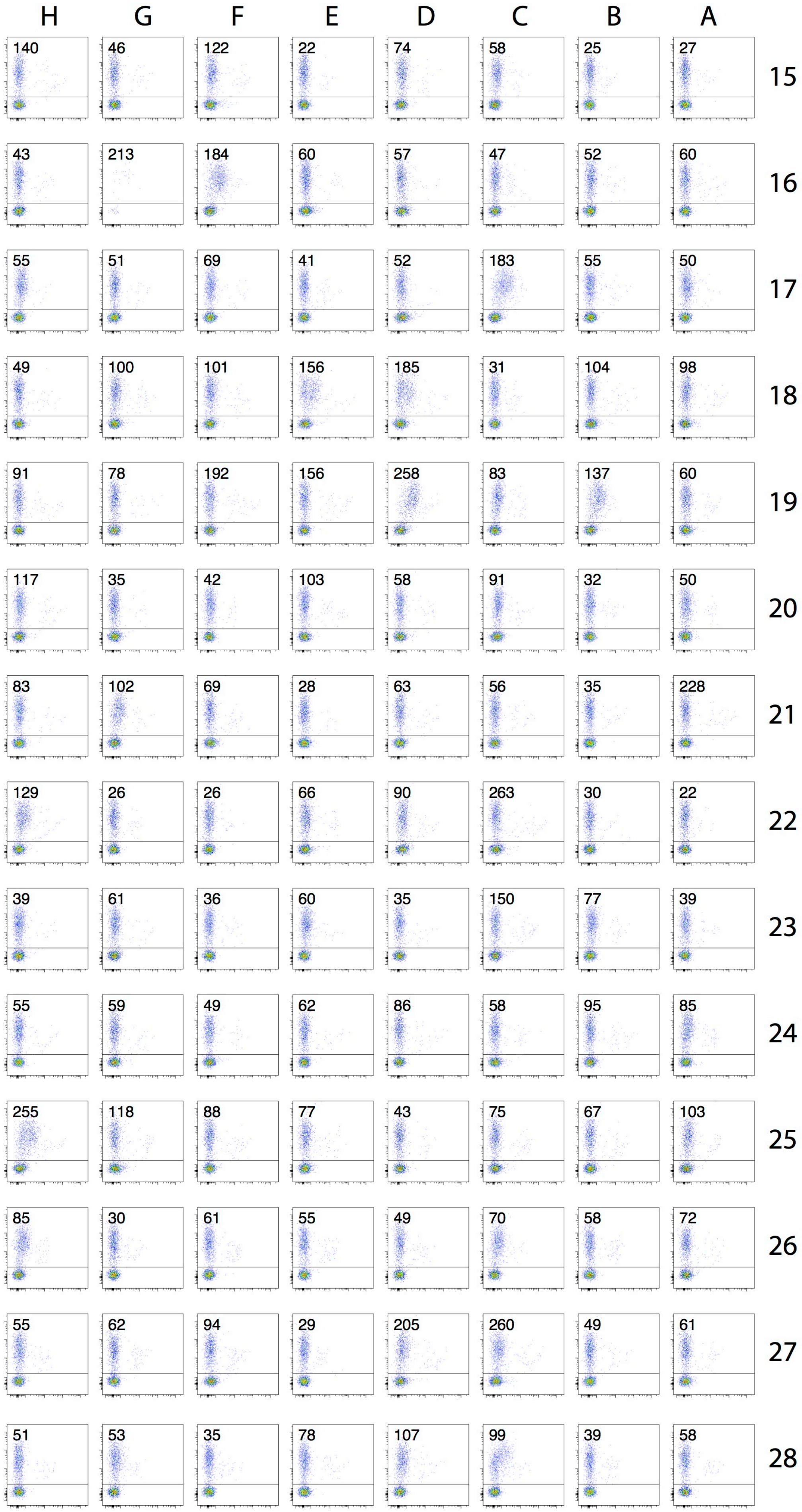
H G F E D C B A
140 46 122 22 74 58 25 27 15
43 213 184 60 57 47 52 60 16
55 51 69 41 52 183 55 50 17
49 100 101 156 185 31 104 98 18
91 78 192 156 258 83 137 60 19
117 35 42 103 58 91 32 50 20
83 102 69 28 63 56 35 228 21
129 26 26 66 90 263 30 22 22
39 61 36 60 35 150 77 39 23
55 59 49 62 86 58 95 85 24
255 118 88 77 43 75 67 103 25
85 30 61 55 49 70 58 72 26
55 62 94 29 205 260 49 61 27
51 53 35 78 107 99 39 58 28

Supplementary Table I.
Estimated material costs for the FACS assay (calculated for a plate with 96 samples)

**materials**	**costs (€)**
cell culture flask, 96 well plate	1.5
FACS tubes	5
cell culture medium	0.15
fetal bovine serum	0.15
plasmid preps	0.2
polyethylenimine	0.1
PBS	1
bovine serum albumin	0.5
PE-conjugated secondary Ab	10
**total**	**18.6**

Supplementary Table II
Levels of SARS-CoV-2-S-specific antibodies in healthy coworkers at the University Medical Center Hamburg during the epidemic.

ID	age	IFM	FACS	Plate
1	22	-	1,0	1C04
2	23	-	0,6	1B04
3	23	-	0,5	1E05
4	23	-	0,6	1E10
5	23	-	0,5	1F04
6	23	-	0,8	1H05
7	25	-	0,6	1G04
8	25	-	0,7	1H04
9	26	-	0,8	1A03
10	26	-	0,5	1F05
11	30	-	0,6	1D05
12	33	-	0,6	1D04
13	37	-	0,8	1G05
14	38	-	0,7	1G10
15	43	-	0,6	1E04
16	56	-	2,3	1A04
17	58	-	0,8	1H10

Supplementary explanations for Table II

Patient H1-1, a physician at our hospital, acquired SARS-CoV-2 in Trentino, Italy. After his return to Hamburg on February 23rd he transmitted CoV-2 to two of his 132 contacts, including his wife (individual H1-2) (1). Members of families H2, H3, and H4 reported fever and headaches on March 5th, 8th, and 9th, respectively. Other family members developed symptoms within the next 10 days. Both members of H2 showed typical symptoms and were tested PCR positive within 3 days of another, both developed antibodies. Family H3 returned from Lech, Austria on March 8th. H3-1 (50-yr) was the first to develop symptoms on March 9th and was tested PCR+ a few days later. H3-2 and H3-3 developed COVID-19 symptoms on March 14th and were also tested PCR positive. All members of family H3 developed antibodies. It is suspected that H3-4 (19-yr) was actually the first one to acquire the disease. He does not recall any symptoms beyond a mild sore throat for 1 day and was tested SARS-CoV-2 negative by PCR on March 15th and on March 26th. He did develop CoV-2-S-specific antibodies, suggesting that he may have been the first member of his family to acquire SARS-CoV-2, but had already eliminated the virus by the time of his PCR-tests. Interestingly, his 21-yr old sibling H3-3 was tested PCR positive and remained PCR+ for more than 4 weeks, despite only weak symptoms. All symptomatic members of family H4 developed antibodies with one exception: H4-2 (26-yr) reported symptoms on March 15th but was not tested by PCR. He did not develop any detectable CoV-2 specific antibodies. In H5, the mother reported fever and headache on March 9th, but her 4-yr old son remained asymptomatic throughout the quarantine. Family H6 represents two close contacts of an 89-yr old patient that contracted COVID-19 in early March while in the hospital for unrelated disease and that succumbed to COVID-19 related complications in early April. H6-1 and H6-2 probably got infected while visiting this patient before the hospitals were closed for visitors. This patient likely was a super-spreader, because seven of the eight relatives that visited him in the hospital contracted COVID-19. H7 and H8 represent households of physicians that acquired SARS-CoV-2 in Hamburg, most likely at work. H7-1 developed symptoms on March 23rd and was tested positive two days later. H8-1 became ill on March 31st and was tested positive by PCR the following day. She reported high fever, headache, and anosmia for three days.

1. Pfefferle S, Kobbe R, Guenther T, Noerz D, Santer R, Oh J, et al. (2020). Infection Control and Virological Assessment of the First Cluster of COVID-19 in Northern Germany. *SSRN* preprint. doi: doi.org/10.2139/ssrn.3572733 PREPRINT.
